# Arteriojejunal Fistula Presenting with Recurrent Obscure GI Hemorrhage in a Patient with a Failed Pancreas Allograft

**DOI:** 10.1155/2013/171807

**Published:** 2013-12-25

**Authors:** Nirmit Desai, Sagar Patel, Chinyere Nwosu, Lok Sung, Carl Tack, Jonathan M. Buscaglia, Edward P. Nord, Nand K. Wadhwa

**Affiliations:** ^1^Department of Medicine, Stony Brook Medicine, Stony Brook, NY 11794, USA; ^2^Division of Nephrology, Department of Medicine, Stony Brook Medicine, Stony Brook, NY 11794, USA; ^3^Divisions of Gastro-Enterology, Department of Medicine, Stony Brook Medicine, Stony Brook, NY 11794, USA; ^4^Department of Radiology, Stony Brook Medicine, Stony Brook, NY 11794, USA

## Abstract

We present a case of a patient with a failed pancreaticoduodenal allograft with exocrine enteric-drainage who developed catastrophic gastrointestinal (GI) hemorrhage. Over the course of a week, she presented with recurrent GI bleeds of obscure etiology. Multiple esophago-gastro-duodenoscopic (EGD) and colonoscopic evaluations failed to reveal the source of the hemorrhage. A capsule endoscopy and a technetium-labeled red blood cells (RBC) imaging study were similarly unrevealing for source of bleeding. She subsequently developed hemorrhagic shock requiring emergent superior mesenteric arteriography. Run off images revealed an external iliac artery aneurysm with fistulization into the jejunum. Coiled embolization was attempted but abandoned because of hemodynamic instability. Deployment of a covered endovascular stent into the right external iliac artery over the fistula site resulted in immediate hemodynamic stabilization. A high index of suspicion for arterioenteric fistulae is needed for diagnosis of this uncommon but eminently treatable form of GI hemorrhage in this patient population.

## 1. Introduction

Simultaneous pancreas and kidney transplantation is the standard of care for type 1 diabetes mellitus (DM) with end-stage renal disease (ESRD) with an increased frequency of exocrine enteric drainage [1]. Common vascular complications after pancreas transplantation include venous and arterial thrombosis, vascular stenosis and kinks, and arterial dissection due to either clamping injuries or native atherosclerotic disease [2]. Uncommon vascular complications after pancreas transplantation include arteriovenous (AV) fistulae and pseudoaneurysms, arterioenteric (AE) fistulae, enteric anastomotic bleeding, and mycotic pseudoaneurysms [3–10]. AE and AV fistulae after failed pancreas allograft are challenging entities for obscure GI bleeding. Bleeding from these AE/AV fistulae can be extensive leading to increased morbidity and mortality [6, 7, 11]. The diagnosis of these vascular abnormalities is often perplexing, and a high index of suspicion is required in patients with previous abdominal operations or radiation who present with overt GI bleeding of uncertain etiology [12–15]. However, catastrophic bleeding in a critically ill patient with an AE/AV fistula following failed pancreas allograft needs urgent embolization and/or endovascular stent placement. We here present a rare case of failed deceased donor pancreas allograft (exocrine enteric drainage and systemic venous system drainage) with recurrent obscure GI bleeding. Ultimately, an external iliac artery aneurysm bleeding into the jejunum was identified on angiography. Hemorrhage was controlled by deployment of an endovascular stent without requiring surgical intervention.

## 2. Case Report

A 54-year-old woman presented to the emergency room of a community hospital with hematochezia and lightheadedness. Her past medical history was significant for end-stage renal disease (ESRD) due to type 1 diabetes mellitus (DM), for which she had undergone deceased donor kidney and pancreas transplant in 1989. The exocrine pancreas drained into the bladder. The pancreas failed and she underwent pancreatectomy in 2006. The kidney failed soon thereafter. She preemptively received a second kidney allograft from a friend and a deceased pancreas allograft (exocrine enteric drainage, and systemic venous system drainage) in 2008. The second pancreas failed in February 2011 when she once more became insulin dependent. Diffuse large B-cell lymphoma was diagnosed in 2009 for which she underwent R-CHOP chemotherapy and rituximab along with a decrease in immunosuppression. She has remained in remission with negative positron emission tomography (PET) scans.

At the time of admission, her home medications included tacrolimus, prednisone, furosemide, omeprazole, and insulin. Physical examination revealed a blood pressure of 115/57 mmHg, heart rate of 75 beats per minute and regular, and temperature of 98.4°F. The abdomen was soft, nontender with multiple incision scars, and a nontender left lower quadrant kidney allograft. Laboratory data revealed a white blood cell count of 4.0 k/mcL, hemoglobin 9.1 gm/dL, hematocrit 26.1%, and platelet count 208 k/mcL. Serum sodium was 145 mEq/L, potassium 4.1 mEq/L, chloride 111 mEq/L, and bicarbonate 27 mEq/L. Blood urea nitrogen was 16 mg/dL, creatinine 0.81 mg/dL, and glucose 145 mg/dL. Serum calcium was 8.6 mg/dL, phosphorus 3.7 mg/dL, and magnesium 1.6 mg/dL. Fecal occult blood tested positive. Esophagoduodenoscopy (EGD) and colonoscopy were performed which failed to reveal a source of gastrointestinal (GI) bleeding. She was transfused two units of packed red blood cells and subsequently discharged home.

Two days after discharge, she experienced episodes of coffee ground emesis and was admitted to another hospital. On this occasion hemoglobin was 7.7 g/dL. EGD was performed which again was inconclusive. After transfusion of 2 units of packed red blood cells (PRBCs), she was transferred to our hospital for further evaluation. When seen, BP was 115/56 mmHg, heart rate was 79 beats per minute and regular, and temperature of 98°F. Heart and lung examinations were unremarkable. The abdomen was soft, nondistended, with no tenderness over the graft. No peripheral edema was noted. A capsule endoscopy was undertaken, which showed a small nonbleeding arterial-venous malformation (AVM) in the duodenum, gastritis, but no blood in the stomach. At this time, hemoglobin was 9.3 g/dL, BUN 19 mg/dL, and creatinine 0.9 mg/dL.

On day 2 after transfer, she had two bloody bowel movements with a concomitant drop in hemoglobin to 7.6 g/dL and received 2 additional units of PRBCs. Repeat colonoscopy was technically limited due to poor bowel preparation but no active bleeding was detected. Later that day, she underwent a technetium-labeled RBC imaging scan which failed to reveal active gastrointestinal bleeding. On day 3 after transfer, she underwent repeat colonoscopy which again was nondiagnostic. Following colonoscopy, she became hypotensive with a systolic blood pressure of 70 mmHg without signs of active bleeding. Repeat hemoglobin was 9.9 g/dL and she received 2 liters of normal saline. On day 4, the hemoglobin dropped to 5.9 g/dL and 2 more units of PRBCs were administered. On day 5, she developed slurred speech and became unresponsive. She was intubated for airway protection and arterial-line systolic blood pressure measured 30–50 mmHg. Physical examination now revealed abdominal distension with hypoactive bowel sounds. An oral-gastric (OG) tube aspiration revealed 2,400 mL of bright red blood. Repeat hemoglobin was 4.4 g/dL. She received 5 liters of normal saline, 4 units of PRBCs, and pressure support with intravenous infusion of vasopressin and norepinephrine. An emergent endoscopy at bedside showed no active bleed from the duodenum; however, active bleeding was noticed from the proximal jejunum.

**Figure 1:**
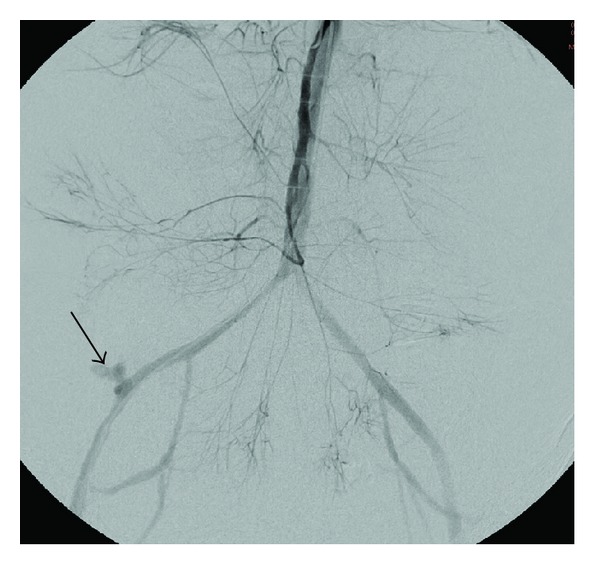
Hand injection of the superior mesenteric artery with reflux into the aorta demonstrates contrast extravasation (arrow) from the right external iliac artery stump, in the region of the patient's pancreatic transplant. Additionally, there is significant mesenteric vasospasm.

**Figure 2:**
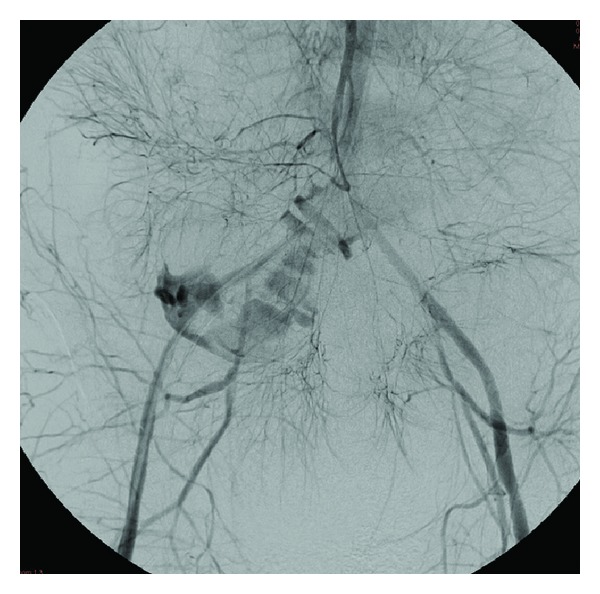
Superior mesenteric arteriogram (with injector device) demonstrates arteriojejunal fistula via the right external iliac artery.

**Figure 3:**
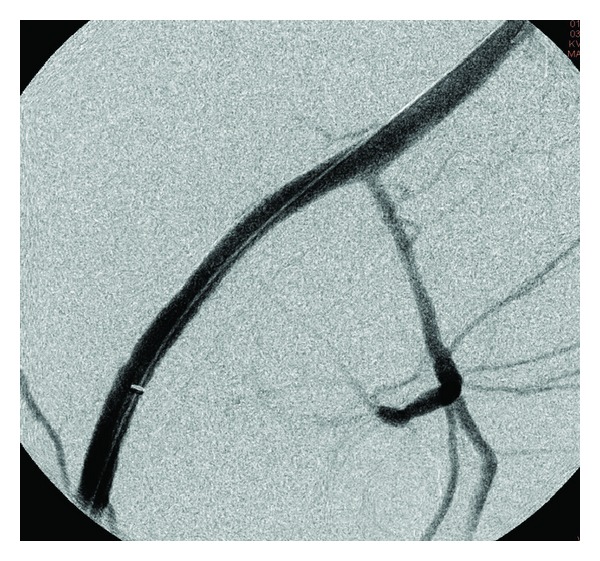
Right common iliac angiogram poststenting demonstrates successful treatment of arterio-jejunal fistula.

Emergent angiography with hand injection of contrast into the superior mesenteric artery was executed. Run off images revealed intense vasospasm and extravasation of contrast medium from the right external iliac artery (Figure 1). Using the injector device, a right external iliac artery aneurysm was identified which communicated with the jejunum (Figure 2). Coil embolization was attempted, but abandoned because of hemodynamic instability. Two iCast 7 mm × 59 mm covered stents (Atrium Medical Corp., Hudson, NH, USA) were deployed into the right external iliac artery over the site of the aneurysm (Figure 3). Normal directional blood flow was instantaneously restored and hemodynamic stability was rapidly attained. Repeat hemoglobin was 12.7 g/dL and serum creatinine was 1.8 mg/dL. The hemoglobin remained stable for the next two days, and no further blood transfusion was required. Of note, during the hospitalization, the patient received a total of 20 units of PRBCs, 14 units of fresh frozen plasma (FFP), 10 units of platelets, 2 units of cryoprecipitate, and 3 units of factor IX.

On day 6 of admission, anuric acute kidney injury became manifested, presumably consequent to ischemic acute tubular necrosis. Continuous renal replacement therapy was instituted for the following 6 days for control of metabolic and volume status. A follow upcomputed tomography (CT) angiogram of the abdomen and pelvis showed a patent right iliac stent with no extravasation on contrast medium. On day 13, the patient was successfully extubated, but remained hemodialysis dependent until day 19 at which point creatinine stabilized at 3.2 mg/dL. On day 20, she was transferred to the hospital where she originally underwent deceased donor pancreas transplantation in 2008. No further intervention was undertaken and the patient was discharged home one week later. Her hemoglobin remained stable at 11.1 g/dL and 11.8 g/dL and serum Cr 3.0 mg/dL and 3.1 mg/dL at the 6, and 12-month followup points, respectively.

## 3. Discussion

We report on a case of recurrent and catastrophic GI bleeding from an unusual arterial-enteric (AE) fistula that developed between an aneurysm of the external iliac artery and jejunum in a patient with a failed pancreatic allograft with exocrine enteric-drainage. Repeated EGD's, colonoscopies, and technetium-labeled RBC imaging failed to reveal the source of GI bleeding. The run off from an abdominal angiogram revealed the site of the AE fistula. Deployment of a covered stent into the right external iliac artery over the site of the aneurysm and fistula restored immediate hemodynamic stabilization and cessation of GI bleeding. No subsequent GI bleeding occurred for the next 12 months.

Both AE and AV fistulae after pancreas transplantation can present as intermittent late lower GI bleeding [16]. Various causes of catastrophic GI hemorrhage after pancreas transplantation include AE and AV fistulae with pseudoaneurysms, aortoenteric fistulae, and enteric anastomotic bleeding [2, 6, 8–11, 17]. AE and AV fistulae can involve the superior mesenteric artery and vein with aneurysmal dilatation and duodenum graft [12], gastroduodenal artery [18], the transplanted arterial graft and the duodenum graft [7], and the graft-recipient arterial anastomosis and stump leak [7] after pancreas kidney transplantation. In the case reported herein, the AE fistula occurred between an external iliac artery aneurysm and the jejunum in the setting of a failed pancreas allograft, leading to life threatening GI hemorrhage. The pathophysiology of such vascular abnormalities appears to be related to inflammation at the site of graft implantation and leakage of pancreatic secretions in this region.

Close monitoring with serial imaging in patients with nonfunctioning pancreatic allografts should be considered so as to detect early fistula formation, and to prevent catastrophic bleeding consequences [19]. Preemptive allograft removal may be considered in certain cases [19]. When catastrophic GI bleeding does occur, as detailed in the patient described herein, endovascular embolization and/or endovascular stent placement should be the treatment of choice. Surgical removal of the failed pancreatic allograft should be considered for the best long-term outcomes, since the incidence of rebleeding among these patients can be high [6, 9].

In summary, we present a case of a patient with a failed pancreas allograft who developed an AE fistula between an iliac artery aneurysm and the jejunum, with a near fatal outcome. In such settings of hemodynamic instability, deployment of an endovascular stent should be considered as the treatment of choice. While there is no guidance from the literature, consideration should be given as to whether a course of antibiotic therapy may be judicious in the setting of an endovascular stent.
